# The genetic basis and the diagnostic yield of genetic testing related to nonsyndromic hearing loss in Qatar

**DOI:** 10.1038/s41598-024-52784-z

**Published:** 2024-02-20

**Authors:** Shaza Alkhidir, Karen El-Akouri, Nader Al-Dewik, Houssein Khodjet-El-khil, Sarah Okashah, Nazmul Islam, Tawfeg Ben-Omran, Mashael Al-Shafai

**Affiliations:** 1 Department of Biomedical Sciences, College of Health Sciences, QU Health, Qatar University, Doha, Qatar; 2 Department of Adult and Pediatric Medical Genetics, Hamad Medical Corporation, Doha, Qatar; 3 Division of Genetic and Genomic Medicine, Sidra Medicine, Doha, Qatar; 4 Department of Public Health, College of Health Sciences, QU Health, Qatar University, Doha, Qatar; 5 Department of Health Research Methods, Evidence and Impact, McMaster University, Hamilton, Ontario Canada; 6 Biomedical Research Center, Qatar University, Doha, Qatar

**Keywords:** Clinical genetics, Consanguinity, Genetics

## Abstract

Hearing loss is the most predominant sensory defect occurring in pediatrics, of which, 66% cases are attributed to genetic factors. The prevalence of hereditary hearing loss increases in consanguineous populations, and the prevalence of hearing loss in Qatar is 5.2%. We aimed to investigate the genetic basis of nonsyndromic hearing loss (NSHL) in Qatar and to evaluate the diagnostic yield of different genetic tests available. A retrospective chart review was conducted for 59 pediatric patients with NSHL referred to the Department of Adult and Pediatric Medical Genetics at Hamad Medical Corporation in Qatar, and who underwent at least one genetic test. Out of the 59 patients, 39 were solved cases due to 19 variants in 11 genes and two copy number variants that explained the NSHL phenotype. Of them 2 cases were initially uncertain and were reclassified using familial segregation. Around 36.8% of the single variants were in *GJB2* gene and c.35delG was the most common recurrent variant seen in solved cases. We detected the c.283C > T variant in *FGF3* that was seen in a Qatari patient and found to be associated with NSHL for the first time. The overall diagnostic yield was 30.7%, and the diagnostic yield was significantly associated with genetic testing using *GJB2* sequencing and using the hearing loss (HL) gene panel. The diagnostic yield for targeted familial testing was 60% (n = 3 patients) and for gene panel was 50% (n = 5). Thus, we recommend using *GJB2* gene sequencing as a first-tier genetic test and HL gene panel as a second-tier genetic test for NSHL. Our work provided new insights into the genetic pool of NSHL among Arabs and highlights its unique diversity, this is believed to help further in the diagnostic and management options for NSHL Arab patients.

## Introduction

Hearing loss (HL) is the most predominant sensory defect worldwide^1^, in which 8% of the cases occur in children^2^. In 2019, 1.5 billion people worldwide were diagnosed with HL^3^. The prevalence of HL in Qatar was estimated in 2005 to be 5.2%^4^. HL can be classified based on its etiology into hereditary hearing loss (HHL) and acquired HL^5^. Overall, HHL accounts for 50–60% of the HL cases^6,7^. Among the cases of childhood-onset HL, around 66% are due to genetic factors^8^. HHL can be isolated, known as nonsyndromic hearing loss (NSHL)—representing around 70% of HL cases^9,10^-, or it can co-exist with other distinctive symptoms and referred to as syndromic hearing loss (SHL). Generally, HHL is genetically heterogeneous, with more than 6000 causative variants reported in at least 150 genes^11^, most commonly in *GJB2* gene. NSHL represents the most significant portion of HHL cases and its associated with pathogenic variants in more than 90 genes^12^. NSHL can be inherited in different modes: 80% of cases are autosomal recessive (AR), 15% of cases are autosomal dominant (AD), and 1–2% of cases are inherited in an X-linked (XL) or mitochondrial pattern^13,14^.

As per the guidelines of the American College of Medical Genetics and Genomics (ACMG)^16^, clinical assessment is usually made through the collection of audiometric data and clinical symptoms as a first step. Secondly, acquired HL is ruled out, and if it cannot be ruled out confidently, evaluation of HHL is made through the appropriate genetic testing. If SHL is suspected, genetic testing specific to the suspected syndrome is performed. In contrast, if NSHL is suspected, single-gene testing of *GJB2* and *GJB6* is conducted as first-tier. If negative or inconclusive, more comprehensive genetic testing such as a HL gene panel or whole exome sequencing (WES) are considered^17^.

Regarding the current knowledge about genetics of HL in the Arab region, a systematic review on HHL reported 104 variants in 44 genes in 17 Arab countries. Of those 104 variants, 20% were found in *GJB2* gene, with the variant c.35delG in *GJB2* gene being the most common, reported in half of the Arab countries. Of all the captured variants, 56 variants were found to be unique to Arabs and associated with variable clinical presentations. Of those 56 variants, 12 variants were reported in patients from Qatar^18^. Additionally, some studies discussed HHL in Qatar and highlighted the high consanguinity rate (51%) and its association with HHL^4,19^. In addition, those studies reported minor contribution of *GJB2* and *GJB6* variants in HHL^20^, and highlighted the important contribution of HL gene panels in identifying the genes and variants associated with NSHL^21^. Four novel NSHL variants were identified in the population of Qatar: c.6614C > T in *CDH23* gene^21^, c.1588G > T in *LOXHD1* gene^19^, c.453_455delCGAinsTGGACGCCTGGTCGGGCAGTGG in *MYO15A* gene^19^, and c.7873T > G in *BDP1* gene^22^.

In Qatar, and like many other countries, the uptake and status of genetic testing in general is variable. In a recent study assessing the attitude towards genetic testing in the Arab region and particularly in Qatar, participants had an overall positive attitude towards genetic testing and expressed their willingness to undergo genetic testing. Furthermore, many factors were found to contribute to such decision including basic knowledge about genetics, past exposure to genetic testing, and a positive family history for a genetic condition^23^. In Qatar, clinical genetics and genomic medicine practice is expanding drastically, along with the availability of a wide range of genetic tests and genetic counseling services. This growth has led to the identification of novel genetic causes for various genetic disorders in Qatar^21,24,25^, though, there is limited literature reporting such findings especially in the context of HHL.

The current study aimed to further explore the spectrum of genetic variation associated with NSHL in the population of Qatar and to assess the diagnostic yield of the various genetic tests offered in the clinical setting.

## Materials and methods

### Hamad medical corporation scope of practice

At Hamad Medical Corporation (HMC), the main health care hospital in Qatar, clinical approach to genetic testing for HL aligns with the ACMG recommendations previously mentioned: *GJB2* gene sequencing is offered as first-tier genetic testing with or without chromosomal microarray, and HL gene panel or WES are offered as second-tier genetic workup. First-tier genetic workup is conducted in HMC local laboratory except for *GJB6* gene testing, which is performed in other laboratories abroad like the second-tier genetic workup.

### Study design and participants

A retrospective chart review was conducted in the Department of Adult and Pediatric Medical Genetics in HMC for pediatric patients diagnosed with NSHL. Ethical approval was obtained for this study from the institutional review boards of HMC (MRC-01-21-614) and Qatar University (QU-IRB 1578-E/21). The study was conducted according to the guidelines of the Declaration of Helsinki. The database of the of Adult and Pediatric Medical Genetics Department at HMC contained more than 20,000 entries at the time of this study, included 336 entries for patients diagnosed with HL. Those 366 patients were further screened for eligibility for inclusion in our study based on the following criteria:

- The patient was below the age of 18 years old at the time of diagnosis and referral.
- The patient had at least one genetic test conducted for the diagnosis of NSHL.

Demographic and clinical data were collected from charts, including gender, ethnicity, nationality, family history, consanguinity, age at diagnosis, type of HL, severity, laterality of HL, usage of hearing assisted tools, history of speech delay, learning difficulties, and audiometric results.

### Genetic testing data

Data was extracted from the genetic testing reports of eligible cases starting with the type of genetic test performed: *GJB2* gene sequencing, chromosomal microarray, targeted familial variant testing, HL gene panel (containing 146 nuclear genes and 6 variants in 4 mitochondrial genes related to HL^26^), WES, or mitochondrial genome testing. Pathogenicity scores of variants were obtained following the ACMG and the association for molecular pathology (AMP) guidelines^27^. The cases were then classified into three main categories based on the likelihood of the identified variants to explain the NSHL phenotype:

1. Solved cases with diagnostic findings: cases with pathogenic or likely pathogenic variants in well-established genes for NSHL, with a zygosity status consistent with the disease’s mode of inheritance, and cases with variants of uncertain significance (VUS) that were solved after familial segregation analysis.
2. Uncertain cases: cases with (VUSs) in a well-established NSHL genes, or cases with variants in genes with limited data/role in relation to NSHL pathogenesis, or cases with variants inherited from an unaffected parent with similar zygosity status.
3. Unsolved cases: cases in which no variants were detected, or cases with variants in NSHL related genes but with an inconsistent zygosity status with the disease’s mode of inheritance (*e.g.* a variant is known to cause the disease in homozygous state but was identified in a heterozygous state in the patient), or cases with variants in genes with no established association with NSHL or HL pathogenesis, or cases with benign variants.

### Analysis of findings of uncertain significance

We conducted further investigation on the findings of “Uncertain cases” category by reviewing familial segregation data -when available- to understand whether a certain genotype was segregating with the NSHL phenotype in each family. VUSs that were supported by familial segregation were re-considered in the “Solved cases” category. While if familial segregation was not available or gave inconclusive findings, the variants remained in the “Uncertain cases” category. Moreover, VUSs and uncertain significance CNVs were searched for in published literature including Single Nucleotide Polymorphism Database (dbSNP) and Ensemble. Copy number variants (CNVs) of uncertain significance were initially searched for in the literature, the database of genetic variant (DGV) (http://dgv.tcag.ca/dgv/app/home) and DECIPHER (https://www.deciphergenomics.org/).in order to further understand their role to NSHL pathogenesis.

### Statistical analysis

The diagnostic yield of each test was calculated by dividing the number of solved cases for each test over the total number this test was used. The overall diagnostic yield was calculated by dividing the total number of solved cases by the total number of the utilization for all tests.

Statistical analysis of the different genetic tests (including *GJB2* gene sequencing, chromosomal microarray, HL gene panel, WES, and mitochondrial genome testing) was conducted using Stata, version 16 (StataCorp, College Station, TX). Chi-square tests (or Fisher exact tests for cells with less than five counts) were used, and *P* < 0.05 (2 tailed) was considered statistically significant. Targeted testing of known familial variants was not included in the analysis, as targeted testing is not part of the stepwise genetic testing routinely performed at HMC, but rather applicable only in cases with a previously known genetic diagnosis of NSHL in the family.

### Ethics approval

This retrospective chart review study involving human participants was performed in line with the principles of the Declaration of Helsinki. Approval was granted by the Medical Research Center at HMC (MRC-01-21-614), and Qatar University Institutional Review Board (QU-IRB 1578-E/21).

### Consent to participate

Due to the retrospective nature of the study, Medical Research Center at HMC (MRC-01-21-614), and Qatar University Institutional Review Board (QU-IRB 1578-E/21). waived the need of obtaining informed consent.

## Results

### Patients’ characteristics

**Figure 1:**
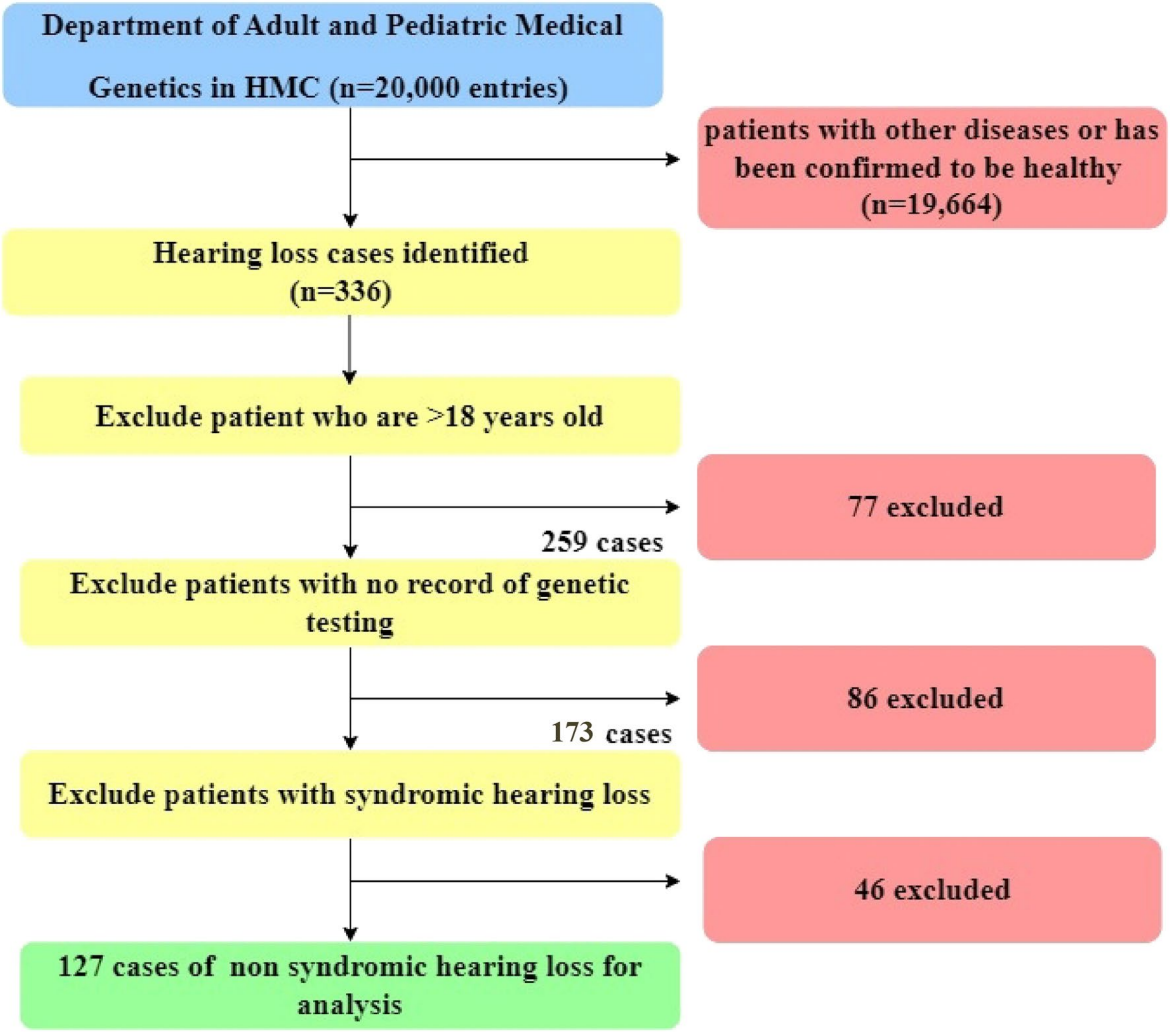
The process of patients screening for the selection of eligible study participants.

A total of 336 cases were referred to the Department of Adult and Pediatric Medical Genetics at Hamad Medical Corporation in Qatar due to HL. After further filtration based on our inclusion criteria, 127 NSHL cases from 100 families were included in our study (Fig. 1).

Sixty-four patients out of 127 were males (50.39%), while 63/127 were females (49.61%). The patients belonged to 19 different nationalities, with most of the patients being from Qatar (39 patients; 30.70%), followed by patients from Pakistan (22 patients; 17.32%), and from Egypt (15 patients; 11.81%). The remaining nationalities are listed in (Table 1). Consanguinity was reported in 79/127 (62.20%) of the cases, and family history of HL presented in 76/127 (59.8%) of our cohort. Sixty-nine out of one hundred twenty-seven (54.3%) of the patients presented with congenital HL (since birth), while 47/127 (37.01%) developed HL later during childhood. Three major types of NSHL were captured, sensorineural hearing loss (SNHL), conductive hearing loss, and auditory neuropathy, with SNHL being the most common type found in 112/127 cases (88.19%). In terms of HL severity, 43/127 (33.86%) patients had severe to profound HL, additionally, 113 patients had bilateral HL (88.98%). The demographic and clinical characteristics of the patients are given in Table 1.

**Table 1:** Demographic and clinical characteristics of the 127 eligible NSHL.

Characteristics	N	(%)
Gender		
Male	64	50.39
Female	63	49.61
Country of origin		
Qatar	39	30.70
KSA	1	0.78
UAE	1	0.78
Yemen	6	4.72
Palestine	6	4.72
Jordan	3	2.36
Syria	8	6.29
India	8	6.29
Pakistan	22	17.32
Iran	1	0.78
Philippines	2	1.57
Egypt	15	11.81
Sudan	6	4.72
Tunisia	3	2.36
Algeria	1	0.78
Ethiopia	1	0.78
USA	2	1.57
UK	1	0.78
Italy	1	0.78
Consanguinity		
Yes	79	62.20
No	41	32.28
Not reported	7	5.51
Family history		
Yes	76	59.84
No	47	37.01
Not reported	4	3.15
Age of onset		
Congenital	69	54.33
Childhood	47	37.01
Not reported	11	8.66
Type of hearing loss		
Sensorineural hearing loss	112	88.19
Conductive	2	1.57
Auditory neuropathy	4	3.15
Not specified	9	7.09
Severity		
Mild to moderate	33	25.98
Moderate to severe	17	13.39
Severe to profound	43	33.86
Progressive	14	11.02
Not reported	20	15.75
Laterality		
Bilateral	113	88.98
Unilateral	13	10.24
Not reported	1	0.79
Usage of hearing tools		
Hearing aid	74	58.27
Cochlear implant	23	18.11
Hearing aid and cochlear implant	12	9.45
No hearing tool	18	14.17
History of speech delay		
Yes	79	62.20
No	32	25.20
Not reported	16	12.59
History of learning difficulties		
Yes	28	22.05
No	34	26.56
Not applicable	30	23.62
Not reported	35	27.56

*KSA* Kingdome Saudi Arabia, *UAE* United Arab Emirates.

### Test frequency and diagnostic yield

*GJB2* gene sequencing was the most utilized test (81.10%) among the five genetic tests. While, among the tests in the second-tier genetic workup category, WES and mitochondrial genome testing were the most utilized tests (23.62% and 15.74% respectively), followed by gene panel (7.87%) and targeted familial variant testing (3.9%) (Table 2).

**Table 2:** The Utilization frequencies of the different genetic tests and their associated diagnostic yields.

Genetic test	Utilization frequency per patients (n = 127)	Solved cases (diagnostic yield)	Uncertain cases	Unsolved cases		*P-Value*
				No Finding	Benign	
*GJB2* gene sequencing	103 (81.10%)	17 (16.5%)	0%	83 (80.6%)	3 (2.9%)	**< 0.001**^**a**^
Chromosomal microarray	65 (51.18%)	1 (1.5%)	4 (6.2%)	57 (87.7%)	3 (4.6%)	0.127^b^
WES	30 (23.62%)	13 (33.3%)	8 (25.8%)	8 (25.8%)	0%	0.573^b^
Mitochondrial genome testing#	20 (15.74%)	0	0	16 (80%)	4 (20%)	0.052^a^
Gene panel	10 (7.87%)	5 (50%)	4 (40%)	1 (10%)	0%	**0.020**^**b**^
Targeted familial variant testing	5 (3.9%)	3 (60%)	0%	2 (40%)	0%	–
Total	–	39 (30.7%)	–	–	–	–

ªFischer test, ^b^Chi Square test. Cut off value is 0.05.

Significant values are in bold.

The overall diagnostic yield was 30.70% (39 solved cases/127 cases). As expected, the highest diagnostic yield per test was achieved by targeted familial variant testing which reached 60% (3/5 cases). This was followed by gene panel, WES, *GJB2* gene sequencing and chromosomal microarray (Table 2). Moreover, two tests were statistically significant in terms of diagnostic yield association, including *GJB2* gene sequencing (*p* < 0.001) and gene panel (*p* = 0.020) (Table 2). The contribution of each genetic test to the overall diagnostic findings yield of the study is given in (Table 2).

**Figure 2:**
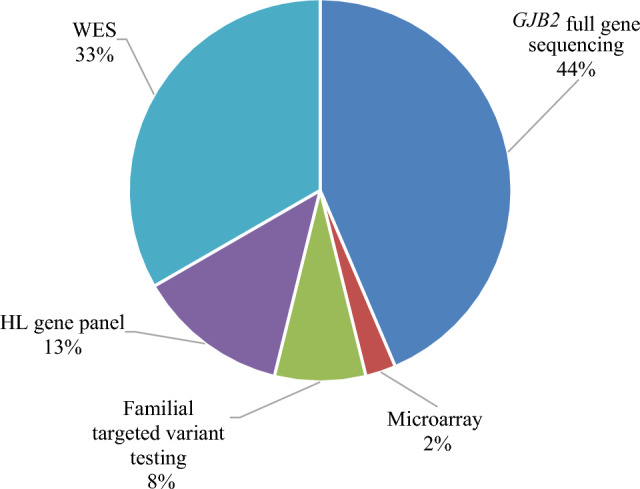
A pie chart showing the contribution of the different genetic tests to the overall diagnostic yield.

The study revealed 50 different variants in 29 genes and 10 CNVs in a total of 59 patients out of 127 patients. 38% of the variants and 20% of the CNVs identified were in the solved cases category (Fig. 2). The largest number of captured variants (22%) were in *GJB2* gene, followed by *OTOF* gene (8%), *MYO15A* gene (6%), *PCDH15* gene (6%), and *TECTA* gene (6%) while the rest of genes accounted for a smaller fraction of variants, ranging between 2 and 4% (Supplementary Fig. 1A). Majority of our patients 44% (56/127) presented with variants in *GJB2,* while 13% of them had a variant in *OTOF* (Supplementary Fig. 1B).

### Variants in the solved NSHL cases

**Figure 3:**
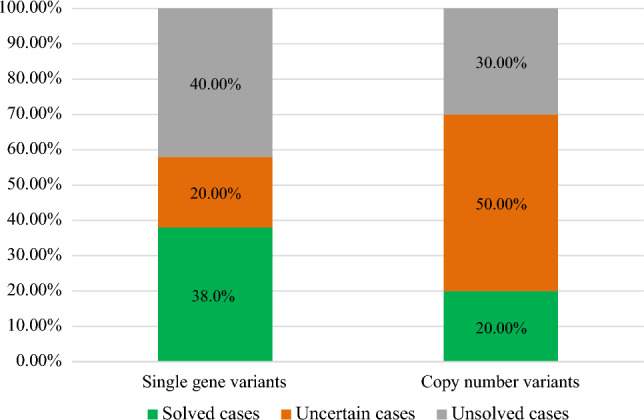
A bar chart illustrating the number of variants (single gene variants and copy number variants) associated with each of the three categories of genetic findings (solved, uncertain, and unsolved).

**Figure 4:**
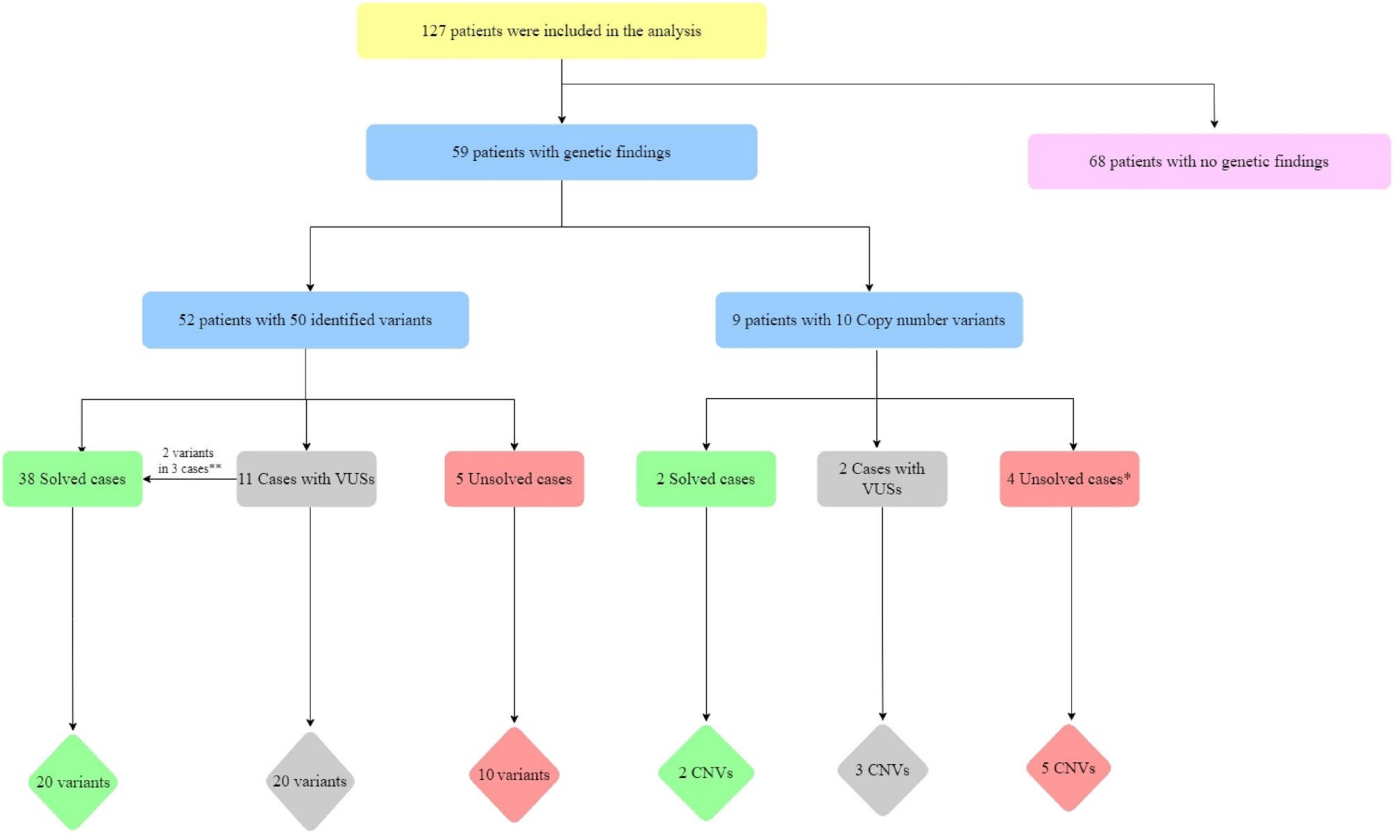
A flowchart summarizing the genetic findings. *One case had unsolved CNV, so it was subjected again to WES and was solved. **subjected to familial segregation.

A total of 19 variants in 11 genes and two CNVs (Fig. 3) explained the NSHL phenotype in the 39 solved cases (Table 3). Around one third of the single gene variants (7 out of the 19 variants) were in *GJB2* gene (Table 3). Based on the initial laboratory report and ACMG-AMP guidelines, 63% of variants (n = 18 variants) were reported to be pathogenic variants as follows: 1 partial deletion in *ABHD12* gene, 7 variants (c.35delG, c.− 23G > T, c.506G > A, c.290dup, c.109G > A, c. − 23 + 1G > A, c.427C > T) in *GJB2* gene*,* 3 variants (c.5375G > A, c.2239G > T, c.1621G > A) in *OTOF* gene*,* one variant* (c.1198delT)* in *SLC26A4* gene, one variant c.92A > G in *TMIE*, c.283C > T in *FGF3*, c.8340G > A in *MYO15A*. Additionally, two likely pathogenic variants were detected, c.1195C > T in *TRIOBP* and c.346G > A in *TMPRSS3*. The remaining variant c.2257 T > C in *ESPN* was initially reported as VUS and then were reclassified based on familial segregation to be in the solved cases category (Fig. 4).

**Table 3:** Variants reported in the solved cases (n = 20).

Gene	Rs ID	Amino acid change	cDNA change	Variant class	Zygosity	Mode of inheritance	Pathogenic score (based on laboratory report)	Testing method	ACMG classification	ACMG sub classification	ClinVar	Patient ID	Age of onset	Country of origin	Phenotype	Familial segregation
ABHD12	–	–	Partial deletion	Deletion	HM	AR	Pathogenic	WES	–	–	–	HL-32	Childhood	Qatar	Moderate SNHL in RT ear and in LT	–
GJB2	rs80338939	p.G12Vfs	c.35delG	Frameshift	HM	AR	Pathogenic	Gene sequencing	Pathogenic	PVS1, PS4, PM1, PP3, BS3	Pathogenic	HL-19	Childhood	Palestine	Bilateral SNHL	–
												HL-27	Childhood	Tunisia	Moderate SNHL in RT ear and severe in LT ear	–
												HL-36	Childhood	Palestine	Severe SNHL in RT ear and moderate in LT ear	–
												HL-38	Childhood	Egypt	Moderate SNHL	–
												HL-53	Childhood	Egypt	Moderate SNHL	–
												HL-62	Childhood	Jordan	Mild SNHL in RT ear and severe to profound LT ear	–
												HL-66	Congenital	Palestine	Severe to profound SNHL	–
												HL-78	Congenital	Tunisia	Mild to moderate SNHL	–
												HL-113	Congenital	Ethiopia	Severe to profound SNHL	–
	rs80338940	–	c. − 23 + 1G > A	Intronic	HM	AR	Pathogenic	Gene sequencing	Likely Pathogenic	PM2, PS4	Likely pathogenic	HL-59	Childhood	Syria	Moderately severe SNHL	–
												HL-65	Congenital	Pakistan	Progressive SNHL	–
												Hl-81	Congenital	India	Mild SNHL	–
												HL-1	Congenital	Qatar	Mild SNHL	–
	rs774518779	p.C169Y	c.506G > A	Missense	HM	AR	Pathogenic	Gene sequencing	Likely Pathogenic	PS3, PP3, PP5	Pathogenic	HL-30	Congenital	Qatar	SNHL	–
												HL-39	Congenital		Severe to Profound SNHL	–
												HL-89	Childhood		Profound SNHL	–
	rs786204491	p.Tyr97*	c.290dup	Stop gained	HM	AR	Pathogenic	Gene sequencing	Pathogenic	PM2, PM1, PVS1	Pathogenic	HL-40	Congenital	Iran	Severe to profound SNHL	–
	rs72474224	p. V37I	c.109G > A	Missense	HM	AR	Pathogenic	Gene sequencing	Likely Pathogenic	PS3, PP3, PP5	Pathogenic	HL-123	Congenital	Philippines	Mild SNHL	–
	rs786204734	–	c. − 23G + 1 > T	Splice donor variant	HM	AR	Pathogenic	Gene sequencing	Pathogenic	PVS1, PS3, PM3, PS4	Pathogenic	HL-121	Childhood	Qatar	Mild SNHL in RT ear and moderate in LT ear	–
																–
OTOF	rs111033349	p.R1792H	c.5375G > A	Missense	HM	AR	Pathogenic	Panel	Likely Pathogenic	PM1, PM2, PP3, PP5	Likely pathogenic	HL-46	Congenital	Qatar	SNHL (type undefined)	–
	rs397515591	p.E747X	c.2239G > T	Nonsense	HM	AR	Pathogenic	WES/Panel	Pathogenic	PVS1, PM2, PP3, PP5	Pathogenic	HL-50	Congenital	Qatar	Auditory Neuropathy	–
												Hl-67	Congenital	Qatar	Severe SNHL	–
												HL-120	Congenital	Sudan	Severe to Profound SNHL	–
	rs397515435	p.G541S	c.1621G > A	Missense	CH	AR	Pathogenic	WES	Likely Pathogenic	PM1, PM2, PP3, PP5	Pathogenic	HL-75	Congenital	Qatar	Auditory Neuropathy	–
SLC26A4	rs397516413	p.C400VfsX32	c.1198delT	Frameshift	HM	AR	Pathogenic	WES/targeted gene sequencing	Pathogenic	PS4, PM1, PM2, PM4	Pathogenic/likely pathogenic	HL-34	Childhood	Syria	Severe SNHL	–
												HL-37	Childhood	Syria	Moderate SNHL in RT and severe in LT ear	–
												HL-116	Childhood	Syria	RT ear is severe to profound, LT ear is moderate SNHL	–
TMIE	rs1057517839	p.E31G	c.92A > G	Missense	HM	AR	Pathogenic	WES	Likely Pathogenic	PM1, PM2, PP3, PP5	Pathogenic	HL-28	Congenital	Qatar	SNHL (type undefined)	–
TRIOBP	rs750078356	p.R399X	c.1195C > T	Missense	HM	AR	Likely pathogenic	WES	Pathogenic	PVS1, PM2, PP5	Pathogenic	HL-61	Congenital	Qatar	Severe to profound SNHL	–
STRC	–	–	–	Deletion	HM	AR	Pathogenic	WES	–	–	–	HL-55	Congenital	Qatar		–
FGF3	rs281860303	p.R95W	c.283C > T	Missense	HM	AR	Pathogenic	WES (trio)	US	PM2, PP5	Likely pathogenic	HL-93	Qatar	Congenital	Severe to profound SNHL	–
GJB2	rs80338948	p.R143W	c.427C > T	Missense	HM	AR	Pathogenic	Gene Sequencing	US	PM2, PP3, PP5, BS2	Pathogenic	HL-17	Egypt	Congenital	SNHL (type undefined)	–
MYO15A	rs878853228	p.T2780 = =	c.8340G > A	Missense	HM	AR	Pathogenic	WES (trio)	US	PM2, PP3	Pathogenic	HL-54	Pakistan	Childhood	Sever to profound SNHL	–
TMPRSS3	rs372526764	p.V116M	c.346G > A	Missense	HM	AR	Likely pathogenic	WES (trio)	US	PM1, PM2, PP5	Likely pathogenic	HL-63	Qatar	Childhood	SNHL (type undefined)	–
Cases that were considered as solved after familial segregation (n = 2)																
ESPN	rs869312937	p.W753R	c.2257 T > C	Missense	HM	AR	US	WES (trio)	US	PM1, PM2, PP3, BP1	Uncertain significance	HL-68	Qatar	Congenital	Severe to profound SNHL	Affected brother shared same variant in HM status
												HL-98		Congenital	Severe SNHL	Affected brother shared same variant in HM status

*HT* heterozygous, *HM* homozygous, *AR* autosomal recessive, *CH* compound heterozygous, *VUS* variant of uncertain significant, *WES* whole Exome Sequencing, *US* uncertain significance, *SNHL* sensorineural hearing loss, *RT* right, *Lt* left.

ACMG classification: PVSI: null variant (nonsense, frameshift, canonical ± 1 or 2 splice sites, initiation codon, single or multiexon deletion) in a gene where LOF is a known mechanism of disease; PM1: Located in a mutational hot spot and/or critical and well-established functional domain (e.g., active site of an enzyme) without benign variation, PM2: Absent from controls (or at extremely low frequency if recessive) in Exome Sequencing Project, 1000 Genomes Project, or Exome Aggregation Consortium; PM4: Protein length changes as a result of in-frame deletions/insertions in a nonrepeat region or stop-loss variants; PP3: Multiple lines of computational evidence support a deleterious effect on the gene or gene product (conservation, evolutionary, splicing impact, etc.); PP5: Reputable source recently reports variant as pathogenic, but the evidence is not available to the laboratory to perform an independent evaluation; PS3: Well-established in vitro or in vivo functional studies supportive of a damaging effect on the gene or gene product; PS4: The prevalence of the variant in affected individuals is significantly increased compared with the prevalence in controls; BP1: Missense variant in a gene for which primarily truncating variants are known to cause disease; BS2: Observed in a healthy adult individual for a recessive (homozygous), dominant (heterozygous), or X-linked (hemizygous) disorder, with full penetrance expected at an early age; BS3: Well-established in vitro or in vivo functional studies show no damaging effect on protein function or splicing; BS4: Lack of segregation in affected members of a family.

*modified ACMG-AMP guideline for mitochondrial variants.

**patient phenotype already explained by other variant in Table 3.

All variants in the solved cases showed an autosomal recessive pattern of inheritance (Table 3). Six variants were in *GJB2* gene among 19 patients including: c.35delG detected in 9 patients, the intronic variant c.− 23G > T in 4 patients, c.506G < A in three patients, along with other three variants (c.290dup, c.109G > A, and c.− 23 + 1G > A) that were detected in three patients respectively (Table 3).

In addition to *GJB2 variants,* three variants were captured in *OTOF* gene (c.5375G > A, c.2239G > T and c.1621G > A), a frameshift variant (c.1198delT) in *SLC26A4*, c.92A > G in *TMIE,* c.1195C > T in *TRIOBP, a* partial deletion in *ABHD12* and a deletion in *STRC.* Details about each variant and patients’ phenotypes are given in Table 3.

### Variants in the unsolved cases

In the unsolved cases category, we identified 10 variants in 6 genes among 5 patients including c.166C > T in *CRYAB*, m.14484T > C in *MT-ND6*, m.12174C > T in *MT-TH*, m.3156A > G in *MT-RNR2*, c.5364-5373del10 and c.*9-*13delTTCTT in *PCDH15*, as well as 4 variants in *GJB2* gene (c.35delG, c.109G > A, c.334_335delAA, and c.487A > G) (Supplementary Table S1). Six of these variants were classified as pathogenic based on the laboratory report, however they could not explain NSHL phenotype due to some reasons such as inconsistent zygosity, or not supported by family segregation (e.g. inheritance from a healthy parent), or the variants were associated with other phenotypes (Supplementary Table S1).

In addition, one benign variant and three VUS were detected. Nevertheless, four variants failed to explain the NSHL phenotype in 3 cases (HL-5, Hl-25, HL-121), however these cases had another identified variants that were able to classify them into either solved or uncertain (Supplementary Table S1,Table 4, Supplementary Table S2). Further details about variants reported in unsolved cases are detailed in Supplementary Table S1.

**Table 4:** Summary of all of the copy number variants observed in NSHL patients cohort (n = 10).

	Cytogenetic band	CNV	Gene included	Zygosity	Mode of inheritance	Laboratory Classification	Parental testing	Testing method	DGV	DECIPHER	Gene involved	Patient ID	Age of onset	Country of Origin	Phenotype of our patients
Solved cases	15q15.3	Duplication of 71 kb	*DFNB16 (STRC), CATSPER2 and CKMT1A*	HM	–	Pathogenic	Not done	Chromosomal Microarray	Not associated	Gain in this region was reported in one case with hearing impairment with other manifestation	Associate with autosomal recessive hearing loss	HL-56	Congenital	Egypt	Moderately severe SNHL
	15q15.3	Deletion of 51 kb	*STRC and CATSPER2* gene	HM	AR	Pathogenic	Not done	Chromosomal Microarray	Not associated	Gain in this region was reported in one case with hearing impairment with other manifestation	STRC associate with mild to moderate hearing loss	HL-107	Childhood	Qatar	Mild to moderate SNHL
Unsolved cases	2p22.1	Deletion of 244 kb	No genes reported in this region	HT	–	Likely Benign	Not done	Chromosomal Microarray	Not associated	Loss in this region was reported in one case with hearing impairment with other manifestation	No association reported to HL	HL-4	Congenital	Palestine	Mild in low frequency SNHL in LT ear
	*2p22*	Duplication of 597 kb	*CRIM1 and FENZ* genes	HT	***-***	Benign	Inherited from a healthy mother	Chromosomal Microarray	Not associated	Gain CNV in this region was reported in two cases with SHL	No association reported to Hearing loss	HL-91	Congenital	India	SNHL (type undefined)
	2p25	Duplication of 493 kb	*SNTG2*				Not done	Chromosomal Microarray	Not associated	Gain CNV in this region was reported in three cases with syndromic sensorineural hearing loss	No association reported to HL	HL-116^a^	Childhood	Syria	Severe to profound SNHL in RT ear and moderate in LT ear
	***2q31.1***	duplication of 53 kb	*KLHL41*	HM	***-***	VUS	Not done	Chromosomal Microarray	Not associated	No reported cases with SNHL	No association reported to HL	HL-57	Congenital	Qatar	Auditory neuropathy
	7p21.2	Deletion 157 kb	*AGMO* gene	HT	–	Likely Benign	inherited from a healthy father	Chromosomal Microarray	Not associated	Loss in this region was reported in one case with hearing impairment with other manifestation	No association reported to HL	HL-125	Congenital	India	Severe to profound SNHL
Cases with uncertain significance findings	9q33.1	Duplication of 740 kb	Not reported	HT	–	VUS	Not done	Chromosomal Microarray	Not associated	Gain in this region was reported in one case with hearing impairment with other manifestation	–	HL-13	Childhood	Egypt	Moderate to severe SNHL in RT ear and severe to profound in LT
	15q13.2	Duplication of 189 kb	Not reported	HT	–	VUS		Chromosomal Microarray	Not associated	Loss in this region was reported in four case with syndromic hearing loss	–				
	Xq13.1	Deletion of 32 kb	EDA gene	HM	–	VUS	Not done	Chromosomal Microarray	Not associated	Loss in this region was reported in five case with non-syndromic hearing impairment and three cases with syndromic hearing loss	Associated with ectodermal dysplasia that might manifest as hearing loss along with other symptoms	HL-115	Childhood	Qatar	severe to profound SNHL

*HT* heterozygous, *HM* homozygous, *VUS*: variant of uncertain significance.

^a^this case was exposed again to WES and found to be solved by a gene variant (check Table 3).

### NSHL cases with uncertain significance variants

Initially we have detected 20 variants in 17 genes among 12 patients that were initially classified as VUSs based on laboratory report (Fig. 3).Two cases were reconsidered as solved cases based on the familial segregation outcomes, including c.2257T > C in *ESPN*. (Table 3)*.* Variant c.2257T > C in *ESPN* was captured in two homozygous unrelated Qatari patients who had one affected sibling, familial segregation revealed than in both cases the affected siblings were also homozygous for the variant c.2257T > C. Additionally, there was one patient with compound variants in *MYO7A* gene, one was a pathogenic variant (c.2476G > A) inherited form a healthy father, and the other was a VUS (c. 4696 A > T), however due to its dual inheritance pattern of both autosomal recessive and autosomal dominant, low penetrance was possible, thus the case was considered as uncertain.

The remaining 20 VUS in 9 patients were kept under uncertain cases category, either because no supportive data from familial segregation were available at the time of the study (Supplementary Table S2). Thirteen variants were detected in heterozygous state in six patients: c.4526A > C in *COL11A1, c.209C* > *T in GJB6,* c.2578T > A in *TECTA,* c.2044C > T in *TJP2,* c.2620G > A in *WFS1,* c.2171G > A in *COL4A4,* c.652_663del12 in *GJB3,* c.680A > G in *MYO3A,* c.*9-*13delTTCTT in *PCDH15,* c.541G > A in *DSCAML1,* c.310T > C in *KCNQ4*. Four variants c.3641G > A and c.6503T > G in *MYO15A*, c.599C > T in *WHRN*, and c.502A > G in *SLC12A2* were captured in homozygous state (Supplementary Table S2). We also identified two compound variants c.− 182G > A.

The case HL-50, had a VUS in *TMPRSS3* gene (c.617-3_617-2dup) and also had a homozygous pathogenic variant in *OTOF* gene. Thus, the case was considered to be solved, however, the exact impact of the VUS in *TMPRSS3* was not fully understood. Further details about these variants are detailed in Supplementary Table S2.

### Copy number variants (CNVs)

We have captured total of 10 CNVs from 9 patients, 2 CNVs solved NSHL phenotype, 4 failed to solve the NSHL phenotype and 4 with uncertain association to NSHL phenotype (Table 4).

The two different CNVs that were found in the solved cases were in the region 15q.15.3 and were classified as pathogenic, one of them was found in the patient as a homozygous gain of 71 kb that falls within the genes *DFNB16 (STRC), CATSPER2,* and *CKMT1A*. The other CNV was a deletion of 51 kb that falls within the genes *DFNB16 (STRC)* and *CATSPER2*. This region was reported in DECIPHER database in multiple cases with hearing loss (Table 4).

Five CNVs were reported in the unsolved cases category. Those CNVs were classified as benign/likely benign or those CNVs had no genes reported to be associated with HL phenotype (Table 4). For example, a Qatari patient with hearing audiopathy was found to be homozygous for a duplication in the region 2q31.1*.* This duplicated region had no association with HL (Table 4). One case (HL-116) was found to have a CNV with unknown clinical significance. This patient underwent WES and was found to have a homozygous pathogenic variant in *SLC26A4* gene, thus the case was considered to be solved (Table 3).

Three CNVs were found to have uncertain association to the NSHL phenotype, all of them were classified as VUS based on the laboratory report (heterozygous duplication in 9q33.1, heterozygous duplication in15q13.2 and a homozygous deletion in Xq13.1). No parental testing has been done to any of them (Table 4). Variations among these regions were reported in DECIPHER database in cases with syndromic hearing loss except for Xq13.1 that was reported with non-syndromic hearing loss too. Details about the CNVs are described furtherly in Table 4. However, due to the lack of parental samples, we could not conclude regarding the involvement of those CNVs in relation to the NSHL phenotype.

## Discussion

### Variant in the solved cases category

The study revealed 50 variants in 29 genes and 10 CNVs captured among 59 pediatric patients with NSHL. Thirty-nine cases (30.7%) were solved due to 19 variants in 11 genes and 2 CNVs. The most common variant was c.35delG in *GJB2,* and it was seen in 9 out of 39 solved cases (22.5%). Initially, 37 patients were found to have either pathogenic or likely pathogenic variants in a gene associated with NSHL. Among the 39 solved cases, 2 patients were initially found to have a VUS and reconsidered as solved cases after the familial segregation results.

At the gene level, third of the identified variants in the solved cases were located in *GJB2*, making *GJB2* the most common gene reported in our pediatric cohort. Historically, variation in *GJB2* was found to be a significant cause of NSHL in different populations, such as in Germans^28^, Northern Europeans^29^, Middle Eastern^30^, and Chinese^31^. In the context of Arab countries, pathogenic *GJB2* variants have also been commonly identified in patients from the UAE (18% of diagnostic findings)^32^, Egypt (14.4% of diagnostic findings)^33^, KSA (10.1% of diagnostic findings)^34^, and Mauritania (9.4% of diagnostic findings)^35^. Our results showed that NSHL patients from Qatari origin had variable scale of variants located in other different genes (*OTOF, TMIE, TRIOBP,* and *TMPRSS3)* along with *GJB2,* which has been already reported in previous reports. Those reports suggested that variations in *GJB2* are not major contributor to NSHL among Qatari patients^19,20^. This can be lent to the possibility of genetic heterogeneity in the Qatari population and the fact that the majority of participants in those previously published studies were of Arab origin or Bedouins^19^, while our study participants were ethnically diverse. This suggests to adapt more comprehensive test options for the NHSL patients in Qatar patients to capture the broad spectrum of causative genes and variants.

At the variant level, c.35delG in *GJB2* gene was the most common variant in our cohort*,* observed in 9 patients out of the 39 solved cases. this result aligns with the previous reports indicating its high frequency among NSHL patients from Algeria^36^, Mauritania^35^, Egypt^37^, and UAE^32^, Kuwait^38^, Tunisia^39^ along with patients from European origin^40^. For instance, in Tunisia, c.35delG was seen in 35% of NSHL patients and accounted for 85.4% of all variants identified in *GJB2* gene^39^. Similarly in Kuwait, c.35delG was seen amongst 80% of patients with *GJB2* variants^38^. Additionally, this variant represents around 66.7% of *GJB2* variants in Europeans NSHL patients^40^. This finding stress on the importance to prioritize investigation c.35delG in patients from Middle Eastern and Southern European origin where it is believed to be a founder mutation^41,42^.

The second most common recurrent variant in solved cases was c.− 23 + 1G > A in *GJB2,* seen in four patients from Qatar, Syria, Pakistan, and India, in homozygous state. This variant was formerly reported as a founder mutation that originated from central Asia and spread over Eurasia and other regions of the world as result of migration^43^. Nowadays, it is known to be the second most reported variants among hearing loss patients from South Asia^44^, especially Iran^45^ Furthermore, this variant is less expressed in other countries such as Syria^46^, Egypt^33^, Palestine^47^, and KSA^48^. In Qatar, 5 patients with the c.− 23 + 1G > A variant in *GJB2* were previously reported to have this variant also in homozygous state, similar to the zygosity state seen in the Qatari patient in our cohort^20^. This bring the attention to further screen among Qatari families with history of NSHL for this specific variant (c.− 23 + 1G > A), for example at premarital stage, due to the recurrence of this recessive variant and the high rate of consanguineous marriages among Qataris^49^.

Furthermore, three homozygous variants (c.2239G > T, 5375G > A, c.1621G > A) in *OTOF* gene were identified in 4 Qatari and one Sudanese patients among our cohort. Overall, *OTOF*-related NSHL is mainly presented with severe to profound NSHL, or with auditory neuropathy. This phenotype-genotype was similar to what we have seen in our 5 cases who had *OTOF* variants. Highest prevalence of variants in *OTOF* gene were reported among the Spanish population (5%-8%)^50^ and to a lesser extent among Japanese (1–2%)^51^ and Pakistani (2–3%)^52^ populations. In Arabs, the prevalence of *OTOF* variants among NSHL Arab patients is still understudied, and the c.2239G > T variant was also reported in a Libyan patient with severe NSHL^50^, and a Qatari patient with severe NSHL^21^. Considering that 4 out 15 Qatari patients from our cohort had a homozygous pathogenic variant in *OTOF*, it is possible that these variants have a higher prevalence than expected among the Qatari population especially when considering the high consanguinity rate in the population. Clearly, this needs further investigations along with c.92A > G in *TMIE* and c.1195C > T in *TRIOBP* that were identified in homozygous state in two Qatari patients, while no previous reports of these variants in the Qatari patients were published before and to our knowledge this the first time to be reported. The variant c.283C > T in *FGF3* was reported in a single Qatari patient in our cohort. Historically, the c.283C > T variant was related to a syndromic form of HL of variable clinical presentation known as congenital deafness with labyrinthine aplasia, microtia, and microdontia also called LAMM syndrome (OMIM 610,706)^53^. However, we report this variant to be associated with NSHL in Qatar for the first time.

Furthermore, three Syrian patients carrying the homozygous variant c.1198delT in *SLC26A4* presented with variable NSHL severity, this variant was previously reported in four Turkish patients with variable severity of hearing loss^54^, and in patients from Iran^55^. Biallelic pathogenic variants in *SLC26A4* gene are well known to be associated with autosomal recessive Pendred syndrome, which is characterized by early onset of hearing loss along with thyroid gland enlargement, and for lesser extent intellectual disability^56^. To our knowledge, none of our patients presented with any other health complaint other than hearing loss; however, the clinical feature of thyroid involvement associated with Pendred syndrome is known to be variable and present in about 50% of patients^57^.

Two cases were reclassified from being uncertain into solved cases based on the familial segregation results including two cases of Qatari patients who harbored the variant c.2257T > C in *ESPN.* This variant has been previously reported in an Emirati family with HL, and considering the ethnical similarities between the Gulf populations, it could be a founder mutation^58^. One of the uncertain cases was for a Pakistani child who had compound heterozygous variants in *MYO7A* gene, one variant was a pathogenic variant from a healthy father, and the other variant was a VUS c.4696A > T. Variants in in *MYO7A* gene has dual mood of inheritance dominant and recessive. *MYO7A* has reported to cause multiple form of hearing loss, including dominant type^59,60^, variable penetrance and expressivity might affect the interpretation of the familial segregation results, especially when there are limited published data about the variant. Given all, the case was still considered uncertain and further study is needed to explore this variant in order to have more confident clinical judgment.

We report two pathogenic CNVs located within region 15q15.3 in two patients from Qatar and Egypt with moderate NSHL. The two CNVs identified encompass the *STRC* and *CATSPER2* genes, one duplication and one deletion, respectively. The CNVs involving *STRC* and *CATSPER2* were reported in the literature as a common CNV associated with congenital mild to moderate NSHL^61,62^ which is similar to our findings. CNVs involving *STRC* gene are considered the most common CNV associated with NSHL, representing almost two-thirds of all CNVs related to NSHL^63^.

### Variants in the unsolved cases category

We report 10 variants and 5 CNVS were found to be less likely to explain NSHL phenotype as some of them are associated with other diseases, or classified as benign, or inherited from an unaffected/asymptomatic parent. Four of these variants (c.35delG, c.109G > A, c.334_335delAA, and c.487A > G) were located in *GJB2* and were classified as pathogenic based on the laboratory report and pathogenic/likely pathogenic based on ACMG-AMP guidelines, except for c.487A > G that was classified as VUS. These variants were excluded as a cause as they were present in a heterozygous state in our patients which is inconsistent with the zygosity status of the *GJB2* gene variants which follow autosomal recessive mode of inheritance. However, the possibility that patients might harbor other deletion or duplications in these genes cannot be excluded as deletion/duplication testing was not performed.

### Variants in the uncertain cases category

We initially captured 27 VUSs and 3 CNVs in this category. Cases with VUSs were not reclassified as familial segregation analysis was not available at the time of study. However, for other variants their zygosity status might support their involvement in NSHL pathogenesis including c.3641G > A and c.6503T > G in *MYO15A* [reported by the Department of Genetics, SQUH-Genetics Sultan Qaboos University Hospital in Oman to cause NSHL in homozygous state (53)], c.599C > T in *WHRN*, c.2476G > A and c.4696A > T in *MYO7A* (compound heterozygous), c.− 182G > A and c.617-3_617-2dup in *TMPRSS3* (compound heterozygous), and c.98G > A in *OTOF*. Moreover, two of those variants, c.599C > T in *WHRN* and c.98G > A in *OTOF* indicated a damaging impact on the protein structure.

Three VUS CNVs were with limited information such as 9q33.1 and 15q13.2, which were captured in one patient from Egypt, in which the genes involved are unclarified however few cases reported in DECIPHER with copy numbers in these regions manifested with NSHL. Another CNV of uncertain significance is Xq13.1, which involves *EDA* gene that is associated with ectodermal dysplasia, a group of abnormalities that might manifest with hearing loss; however, our patient had NSHL with no other complications^64^. Further investigation, reports, and functional analysis are needed in order to have a comprehensive view about these variants and CNVs in their possible contribution to NSHL phenotypes.

### Test utilization and diagnostic yield of different genetic tests

*GJB2* gene sequencing and chromosomal microarray had the highest utilization rate, estimated at 80.5% and 50.8%, respectively. This could be attributed to the fact that these two tests are conducted locally at HMC as a first-tier workup and free of charge for residents and citizens of Qatar. The overall diagnostic rate in our cohort was 30.7%. diagnostic yield was variable in previous studies, for example a study reported in Germany estimated the diagnostic yield to be 25%^65^while other studies estimated it to be 66%^66^ which could be attributed many factors including variation in the study cohorts, the available tests and their utilization. As expected, the highest diagnostic yield (60%) was obtained using targeted familial testing that relies on the presence of a known causative variant for NSHL in the family. Following targeted familial testing, the second highest diagnostic yield (50%) was obtained using gene panel of 146 nuclear genes and 6 variants in 4 mitochondrial genes related to HHL. Previous studies from Qatar reported diagnostic yield of 50% using a panel of 81 genes for HHL^22^ and a diagnostic yield 33% using a panel of 96 genes for HLL^67^. This similarity could be attributed to the similarity of the genetic background of the cohorts in the two studies as well as the similarity of the core HL genes included in the panel.

Comprehensive genetic tests used for the genetic diagnosis of HHL, such as gene panel and WES, are associated with an increased diagnostic yield, ranging from 10 to 83%, with an average of 41%^68^. Those comprehensive genetic tests provide the highest sensitivity and specificity compared to single gene testing and account for the ethnic variation associated with HHL^69^.

In our cohort, two tests were found to have significant association with diagnostic rate: *GJB2* gene sequencing, gene panel. *GJB2* gene sequencing and gene panel were positively associated with diagnostic yield. Based on our findings, we recommend using *GJB2* gene sequencing, as a first-tier genetic test and gene panel as second tier.

Given our above results, we encourage the current practice of genetic testing for NSHL that is being followed at HMC, as the current practice aligns with the ACMG recommendations and has an overall high diagnostic rate in our population. Furthermore, regarding the second-tier genetic testing, gene panel was found to be statistically significant when compared to WES in our cohort, thus in case where clinicians are indecisive about the choice of second tier-genetic testing, considering panel might be of has higher odds of identifying a genetic etiology. The identification of the underlying genetic cause of NSHL could have an important clinical benefit including offering reproductive options to the couples such as preimplantation genetic testing for monogenic/single gene defects (PGT-M) and/or prenatal testing.

Furthermore, some of the common variants that were specific to the Qatari population such as the variant in *OTOF* gene could be used in the context of premarital screening, where targeted testing may be offered for couples with positive family history for NSHL, especially among consanguineous couples. This will allow the detection of carrier couples for NSHL variants to reduce the burden of the disease, and/or provide couples with appropriate genetic and reproductive counseling.

## Conclusion

In conclusion, we revealed 19 single gene variants in 11 genes and 2 CNVs in 39 patients (30.7%) of our cohort of solved cases with NSHL. Some of these variants were previously reported in NSHL patients while others were reported in our study for the first time. Variants in the *GJB2* gene were the most common genetic cause of NSHL in our population of Qatar, consistent with several studies in many other populations. The *GJB2* variant c.35delG was the most commonly identified among our cohort and it seems to be associated with a less severe presentation of NSHL than in other populations. Our study shed light on one VUSs that seemed to cause of NSHL in our cohort based on family segregation evidence, which merits further investigation and highlights the importance of performing family segregation in the clinical setting when possible. We also recommend using *GJB2* sequencing as first-tier and gene panel as second-tier genetic test for NSHL patients based on their significant association with diagnostic rate in our cohort. Further studies are needed to better understand the pathogenicity of many variants identified in the variants in this study and to reveal the full spectrum of NSHL genes and variants toward developing better diagnostic, management, and treatment options for NSHL.

### Supplementary Information

Supplementary Figure S1.

Supplementary Tables.

## Data Availability

For reasons of privacy and confidentiality, the data from this study are available from the corresponding authors upon reasonable request.
