# Supplementary material for: The genetic basis and the diagnostic yield of genetic testing related to nonsyndromic hearing loss in Qatar
Source: Sci Rep. 2024 Feb 20;14:4202. doi: 10.1038/s41598-024-52784-z (PMC10879212; doi:10.1038/s41598-024-52784-z)
Supplement: Supplementary file 1 — Supplementary Figure S1. [file 41598_2024_52784_MOESM1_ESM.docx]

a

b

Supplementary Figure S1: a. percentage of variants reported in each gene captured within our patient cohort b. number of patients carry variants within genes captured from our patient cohort.
