# Supplementary material for: The genetic basis and the diagnostic yield of genetic testing related to nonsyndromic hearing loss in Qatar
Source: Sci Rep. 2024 Feb 20;14:4202. doi: 10.1038/s41598-024-52784-z (PMC10879212; doi:10.1038/s41598-024-52784-z)
Supplement: Supplementary file 2 — Supplementary Tables. [file 41598_2024_52784_MOESM2_ESM.docx]

| Supplementary table S1: variants that are less likely to explain the non-syndromic hearing loss phenotype among our patient cohort (n=127) | | | | | | | | | | | | | | | | |
| --- | --- | --- | --- | --- | --- | --- | --- | --- | --- | --- | --- | --- | --- | --- | --- | --- |
| ***Gene*** | ***Rs ID*** | ***Amino acid change*** | ***cDNA change*** | ***variant classification*** | ***Zygosity*** | ***Mode of inheritance*** |  | ***Pathogenic score*** | ***Testing Method*** | ***ACMG*** | ***ACMG sub classification*** | ***CLinVar*** | ***Patient ID*** | ***Country of origin*** |  | ***Justification*** |
|  |  |  |  |  |  |  |  | ***(based on laboratory report)*** |  |  |  |  |  |  | ***Phenotype*** |  |
| *GJB2* | rs80338939 | p.G12Vfs | c.35delG | deletion | HT | AR | Pathogenic | | Gene sequencing | Pathogenic | PVS1,PS3,PM1,PP5 | Pathogenic | HL-11 | Syria | LT ear is severe to profound SNHL ( cochlear hypoplasia) | inconsistent zygosity status |
|  | rs72474224 | p.V37I | c.109G>A | Missense | HT | AR | Pathogenic | | Gene sequencing | Likely Pathogenic | PS3,PP3,PP5 | Pathogenic | HL-106 | Philippine | severe to profound SNHL | inconsistent zygosity status |
|  | rs756484720 | p.K112EfrX2 | c.334_335delAA | deletion | HT | AR/AD | Pathogenic | | Panel | Pathogenic | PM2,PVS1,PM3,PP1 | Pathogenic/Likely pathogenic | HL-5 | Egypt | SNHL (undefined type) | Father share same variant with same zygosity but healthy |
|  | rs80338949 | p.M163V | c.487A>G | Missense | HT | AR | pathogenic | | Gene sequencing | uncertain significant | PM2,PP3,BS2,PS3 | Conflicting interpretation | HL-25 | Qatar | LT sever to profound SNHL | inconsistent zygosity status |
| *CRYAB* | rs387907338 | p.R56W | c.166 C>T | Missense | HT | AR/AD | VUS | | WES | Likely Pathogenic | PM1,PM2,PP3,PP5 | Pathogenic | HL-15 | Qatar | SNHL (type undefined) | Associated with other disease( Myopathy and Cardiomyopathy)[1, 2]  (OMIM 123590) |
| *MT-ND6* | rs199476104 | p.M64V | m.14484 T>C | Missense | HOMP | - | Pathogenic | | Mitochondrial testing | USC* | PP7,BS4 | Uncertain significant | HL-122 | Qatar | SNHL in RT ear(microtia and canal atresia) | Associate with other diseases(Leber optic atrophy,MELAS syndrome,Oncocytoma, Parkison disease)[3-6] |
| *MT-TH* | - | - | m. 12174 C>T | Missense | HOMP | - | benign | | Mitochondrial testing | - | - | - | HL-121 | Qatar | RT ear is mild and LT ear is moderate SNHL | Variant is benign, Mother is HOMP and healthy |
| *MT-RNR2* | - | - | m.3156 A>G | Missense | HOMP | - | VUS | | Mitochondrial testing | - | - | - | Hl-45** | Tnisia | Sever to profound SNHL post intratympanic and oral steroids intake | Inherited from a healthy mother with similar homoplasmy |
| *PCDH15* | - | p.P1789LfsX52 | c.5364-5373del10 | deletion | HT | AR | Pathogenic | | Panel | - | - | - | HL-25 | Qatar | Lt ear sever to profound SNHL | inconsistent zygosity status |
|  | - | - | c.*9-*13delTTCTT | deletion | HT | - | VUS | | Panel | - | - | - | HL-45 | Tunisia | Sever to profound SNHL post intratympanic and oral steroids intake | No enough data to establish a diagnosis |

Footnote:

HT:heterozygous, HOMP: homoplasmy, VUS: variant of uncertain significant,WES: whole Exome Sequencing,USC:uncertain significant,SNHL:sensory neural hearing loss,RT:right,Lt:left

ACMG classification:

PM1: Located in a mutational hot spot and/or critical and well-established functional domain (e.g., active site of an enzyme) without benign variation

PM2: Absent from controls (or at extremely low frequency if recessive) in Exome Sequencing Project, 1000 Genomes Project, or Exome Aggregation Consortium

PP3: Multiple lines of computational evidence support a deleterious effect on the gene or gene product (conservation, evolutionary, splicing impact, etc.)

PP5: Reputable source recently reports variant as pathogenic, but the evidence is not available to the laboratory to perform an independent evaluation

PP7: 7 Absent from database, e.g., mtDB and MitoMap, and is heteroplasmic.

BS2: Observed in a healthy adult individual for a recessive (homozygous), dominant (heterozygous), or X-linked (hemizygous) disorder, with full penetrance expected at an early age

BS4: Lack of segregation in affected members of a family

*: modified ACMG-AMP guideline for mitochondrial variants

**:patient phenotype already explained by other variant in Table 3

1. Reilich, Peter, Benedikt Schoser, Nicolai Schramm, Sabine Krause, Joachim Schessl, Wolfram Kress, Josef Müller-Höcker, Maggie C Walter, and Hanns %J Neuromuscular Disorders Lochmuller. "The P. G154s Mutation of the Alpha-B Crystallin Gene (Cryab) Causes Late-Onset Distal Myopathy." 20, no. 4 (2010): 255-59.

2. Brodehl, Andreas, Anna Gaertner‐Rommel, Bärbel Klauke, Simon Andre Grewe, Ilona Schirmer, Andreas Peterschröder, Lothar Faber, Matthias Vorgerd, Jan Gummert, and Dario %J Human mutation Anselmetti. "The Novel Αb‐Crystallin (Cryab) Mutation P. D109g Causes Restrictive Cardiomyopathy." 38, no. 8 (2017): 947-52.

3. Berardo, Andres, Valentina Emmanuele, Wendy Vargas, Kurenai Tanji, Ali Naini, and Michio %J Journal of neurology Hirano. "Leber Hereditary Optic Neuropathy Plus Dystonia, and Transverse Myelitis Due to Double Mutations in Mt-Nd4 and Mt-Nd6." 267, no. 3 (2020): 823-29.

4. Piccoli, Claudia, Maria Ripoli, Giovanni Quarato, Rosella Scrima, Annamaria D’Aprile, Domenico Boffoli, Maurizio Margaglione, Chiara Criscuolo, Giuseppe De Michele, and Annamaria %J Journal of Medical Genetics Sardanelli. "Coexistence of Mutations in Pink1 and Mitochondrial DNA in Early Onset Parkinsonism." 45, no. 9 (2008): 596-602.

5. Ravn, Kirstine, Flemming Wibrand, Flemming Juul Hansen, Nina Horn, Thomas Rosenberg, and Marianne %J European Journal of Human Genetics Schwartz. "An Mtdna Mutation, 14453g→ a, in the Nadh Dehydrogenase Subunit 6 Associated with Severe Melas Syndrome." 9, no. 10 (2001): 805-09.

6. Bartoletti-Stella, Anna, Nunzio CM Salfi, Claudio Ceccarelli, Marcella Attimonelli, Giovanni Romeo, and Giuseppe %J Archives of Ophthalmology Gasparre. "Mitochondrial DNA Mutations in Oncocytic Adnexal Lacrimal Glands of the Conjunctiva." 129, no. 5 (2011): 664-76.

|  | ***Supplementary Table S2:*** variants with uncertain association to non-syndromic hearing loss phenotype among our patient cohort (n=127) | | | | | | | | | | | | | | | | |
| --- | --- | --- | --- | --- | --- | --- | --- | --- | --- | --- | --- | --- | --- | --- | --- | --- | --- |
| ***Gene*** | ***Rs ID*** | ***Amnio Acid change*** | ***cDNA change*** | ***Variant class*** | ***Zygosity*** | ***Mode of inheritance*** | ***Testing method*** | ***Pathogenic score*** | ***ACMG*** | ***ACMG sub classification*** | ***ClinVar*** | ***patient ID*** | ***Country of origin*** | ***Phenotype*** | ***Familial segregation*** | ***ALFA*** | ***gnomAD*** |
| *COL11A1* | rs1057521422 | p.Q1509P | c.4526A>C | Missense | HT | AR/AD | Panel | VUS | USC | PM1,PM2 | VUS | HL-72 | Qatar | Moderate SNHL | Not done | 0.00016 | 0.000007976 |
| *GJB6* | rs727505123 | p.P70L | c.209C>T | Missense | HT | AR/AD | Panel | VUS | USC | PM1,PM2 | VUS | HL-72 | Qatar | Moderate SNHL | Not done | 0.00004 | 0.00001415 |
| *TECTA* | - | p.F860I | c.2578T>A | Missense | HT | AR/AD | Panel | VUS | USC | PM2 | - | HL-72 | Qatar | Moderate SNHL | Not done | - | - |
| *TJP2* | rs760622082 | p.R682W | c.2044C>T | Missense | HT | AR/AD | Panel | VUS | USC | PM1,PM2 | - | HL-72 | Qatar | Moderate SNHL | Not done | 0 | 0.0000349(T) |
| *WFS1* | rs200775335 | p.A874T | c.2620G>A | Missense | HT | AR/AD | Panel | VUS | USC | PM1,PM2,PP3 | VUS | HL-72 | Qatar | Moderate SNHL | Not done | 0 | 0.00006(A) |
| *COL4A4* | rs753659852 | p.R724H | c.2171G>A | Missense | HT | AR/AD | Panel | VUS | USC | PM2,BP4 | VUS | HL-5 | Egypt | SNHL(type undefined) | Not done | 0 | 0.000007(T) |
| *GJB3* | rs727503069 | p.L218_D221del | c.652_663del12 | deletion | HT | AR/AD | Panel | VUS | Likely Benign | BP3,BS1 | Conflicting interpretation | HL-5 | Egypt | SNHL(type undefined) | Not done | 0.001© | 0.0002© |
| *MYO3A* | rs1842309816 | p.D227G | c.680A>G | Missense | HT | AR/AD | Panel | VUS | USC | PM1,PM2 | - | HL-5 | Egypt | SNHL(type undefined) | Not done | - | - |
| *PCDH15* | - | - | c.*9-*13delTTCTT | deletion | HT | - | Panel | VUS | - | - | - | HL-45 | Tunisia | Severe to profound SNHL, post intratympanic and oral steroid intake | Not done | - | - |
| *DSCAML1* | - | p.A181T | c.541G>A | Missense | HT | ND | WES(trio) | ND | USC | PM2,PP2,PP3 | - | HL-25 | Qatar | Lt ear is sever to profound SNHL | Both parents are carrier for the same variant (healthy) | - | - |
| *KCNQ4* | rs866433910 | p.F104L | c.310T>C | Missense | HT | AD | WES(trio) | VUS | USC | PM1,PM2 | - | HL-86 | Qatar | Rt ears is sever to rpofound , LT is moderate to severe SNHL | Mother was confirmed to be carrier but healthy | 0 | - |
| *MYO15A* | rs376676996 | p. R1214Q | c.3641G>A | Missense | HM | AR | WES(trio) | VUS | USC | PM1,PM2 | VUS | HL-111 | KSA | Moderate SNHL | Variant not found in parents | - | 0.000013(A) |
|  | rs1567648703 | p.L2168R | c.6503T>G | Missense | HM | AR | WES(trio) | VUS | Likely Pathogenic | PM1,PM2,PP3,PP5 | Pathogenic | HL-16 | Qatar | Severe to profound SNHL | Mother and sister are healthy heterozygous carriers | 0 | - |
| *WHRN* | rs765757659 | p.T200I | c.599C>T | Missense | HM | AR | WES(trio) | VUS | USC | PP3,PM2 | - | HL-16 | Qatar | Severe to profound SNHL | Mother and sister are healthy heterozygous carriers | 0.0005(A) | - |
| *MYO7A* | rs552367391 | p.T1566S | c.4696A>T | Missense | CH | AR/AD | WES(trio) | VUS | Likely benign | PM2,BS2,BP4 | - | HL-108 | Pakistan | Mild SNHL | Father is a healthy carrier | 0.00031(T) | 0.000014(T) |
| *OTOF* | rs56332208 | p.R33Q | c. 98G>A | Missense | HM | AR | WES(trio) | VUS | Benign | BS1,BS2 | Conflicting interpretation | HL-94 | Qatar | Mild to moderate SNHL | Parents are healthy carriers | - | 0.007(T) |
| *TMPRSS3* | rs147296608 | - | c.-182G>A | Missense | CH | AR | targeted gene sequencing | VUS | USC | PP3,PM2 | VUS | HL-50 | Qatar | Auditory neuropathy | Not done | - | - |
|  | - | - | c.617-3_617-2dup | deletion | CH | AR | targeted gene sequencing | VUS | - | - | - | HL-50 | Qatar | Auditory neuropathy | Not done | - | - |
| *SLC12A2* | - | p.N168D | c.502A>G | Missense | HT | - | WES(trio) | VUS | USC | PM1,PM2,BP1 | - | HL-118 | Qatar | Progressive SNHL | Not found in parents (novel) | - | - |
| *TECTA* | rs200977539 | p.T1891M | c.5672 C>T | Missense | HT | AR/AD | VUS | WES(trio) | USC | PM1,PM2,BS2 | - | HL-25 | Qatar | LT ear sever to profound SNHL | Parents are healthy carriers | 0.00006 | 0.0003763 |
|  | rs727503457 | p. A567T | c.1699 G>A | Missense | HT | AR/AD | VUS | Panel | USC | PM2 | Uncertain significant | HL-82 | Qatar | Mild SNHL | Not done | - | - |

Footnote:

HT:heterozygous,HM:homozygous, AR:autosomal recessive,CH:compound heterozygous, VUS: variant of uncertain significant,WES: whole Exome Sequencing,USC:uncertain significant,SNHL:sensory neural hearing loss,RT:right,Lt:left

ACMG classification:

PM1: Located in a mutational hot spot and/or critical and well-established functional domain (e.g., active site of an enzyme) without benign variation

PM2: Absent from controls (or at extremely low frequency if recessive) in Exome Sequencing Project, 1000 Genomes Project, or Exome Aggregation Consortium

PP3: Multiple lines of computational evidence support a deleterious effect on the gene or gene product (conservation, evolutionary, splicing impact, etc.)

PP5: Reputable source recently reports variant as pathogenic, but the evidence is not available to the laboratory to perform an independent evaluation

PS3: Well-established in vitro or in vivo functional studies supportive of a damaging effect on the gene or gene product

PS4: The prevalence of the variant in affected individuals is significantly increased compared with the prevalence in controls

BP1: Missense variant in a gene for which primarily truncating variants are known to cause disease

BP3: In-frame deletions/insertions in a repetitive region without a known function

BP4: Multiple lines of computational evidence suggest no impact on gene or gene product (conservation, evolutionary, splicing impact, etc)

BS1: Allele frequency is greater than expected for disorder

BS2: Observed in a healthy adult individual for a recessive (homozygous), dominant (heterozygous), or X-linked (hemizygous) disorder, with full penetrance expected at an early age
